# Editorial: Peste des Petits Ruminants (PPR): Generating Evidence to Support Eradication Efforts

**DOI:** 10.3389/fvets.2020.636509

**Published:** 2021-01-27

**Authors:** Francois Louis Roger, Guillaume Fournié, Aurelie Binot, Barbara Wieland, Richard Anthony Kock, Adama Diallo, Alexandre Caron, Bryony Anne Jones

**Affiliations:** ^1^CIRAD, UMR ASTRE, Montpellier, France; ^2^Royal Veterinary College (RVC), University of London, Hatfield, United Kingdom; ^3^International Livestock Research Institute (ILRI), Addis Ababa, Ethiopia; ^4^ISRA-LNERV, Dakar, Senegal; ^5^Faculdade de Veterinária, Universidade Eduardo Mondlane, Maputo, Mozambique

**Keywords:** peste des petits ruminants, epidemiology, ecology, surveillance, control, eradication

Peste des petits ruminants (PPR) is a major transboundary animal disease from a socio-economic point of view. It is also a disease that affects wildlife, threatens susceptible rare wild artiodactyl species and is of conservation concern. In most of Asia and Africa, where the disease is endemic, PPR has a considerable impact on rural economies and the livelihoods of smallholder farmers and pastoralists for whom sheep and goats are often the main assets. In 2015, FAO and OIE developed a global strategy that aims to eradicate PPR by 2030. This strategy heavily relies on large-scale vaccination of sheep and goats, vaccination monitoring and disease surveillance. However, the implementation of this strategy faces several logistical constraints and knowledge gaps, ones which can be addressed through dedicated research programmes. These include: the development of cost-effective thermotolerant vaccines, variations in PPR virus (PPRV) transmission levels in different settings, the structure of networks of contacts between small ruminant flocks and their role in short- and long-range PPR virus dissemination, the impact of small ruminant population dynamics on vaccination frequency and coverage, the roles of wildlife populations and domestic species other than small ruminants (e.g., cattle, camels) in PPR maintenance and spread (and susceptibilities), the socio-economic and biodiversity impacts of PPR disease, efficiency of PPR related animal health services, farmers' perceptions and acceptance of PPR vaccination and their decision making around disease control.

This Research Topic brings together a number of publications on the virology, epidemiology, ecology and control of PPR virus. Gaps in scientific knowledge and ways to enhance control and eradication strategies are also identified. Although PPR is endemic in both Africa and Asia, most publications presented here focus on sub-Saharan Africa, mainly Southern Africa and East Africa, with a few studies on West Africa. Some papers are not geographically tagged as they propose a more broad-based thinking.

Starting with Southern Africa, which is largely free of PPR infection, Mapaco et al. describe a serological survey coupled with event-based surveillance in Mozambique. No evidence of PPR viral circulation was found, despite the disease being endemic in neighboring Tanzania as described by Idoga et al.. Another study by Britton et al. demonstrates the need for enhancing the risk-based surveillance capacity and rapid response in the region, to prevent, and prepare for, possible incursions of PPR.

Multidisciplinary approaches and modeling methods can inform PPR surveillance and control. For instance, spatial modeling of the risk of PPR occurrence based on multicriteria evaluations was applied by Ruget et al. to East African countries, including nearby Indian Ocean islands. It made it possible to identify areas where PPR surveillance and control should be strengthened.

The identification of areas and farming systems which should be targeted by risk mitigation interventions was also the objective of another study carried out by Nkamwesiga et al. who combined participatory epidemiology, classical disease surveillance methods, and genetic analysis in Uganda and along its borders. The results highlight the need of transboundary interventions. This was also emphasized by molecular analyses of PPRV strains collected in West Africa. Indeed, the study by Tounkara et al. and Tounkara et al. suggests frequent transboundary spread of the virus, supporting the need for the genetic characterization of PPRV strains at an international scale, in order to better understand PPRV spatial dynamics and to adapt control measures accordingly.

Mathematical modeling is crucial to optimize vaccination strategies as shown by ElArbi et al., but engagement with farmers and other relevant stakeholders is also essential, as illustrated in Mali by Dione et al.. An important element in the planning of vaccination campaigns is also the willingness to vaccinate, and in places where vaccination is not provided free of charge, also the willingness to pay—both of which depend on different factors as investigated by Wane et al..

Uncertainties regarding the actual host range of PPRV could threaten the effectiveness of the current eradication strategy. Dou et al. reviewed the scientific literature regarding the potential roles of species other than domestic small ruminants in PPRV ecology and PPR epidemiology. For these authors, further investigations are needed in wildlife and atypical domestic hosts, especially in swine and carnivores. Bovines can become seropositive after infection by PPRV, but there is no evidence that they excrete the virus. Therefore, Agga et al. suggest that cattle could serve as sentinel animals for PPR surveillance as they are not targeted by vaccination programmes.

Fine et al. focus their report on wildlife-livestock socio-ecosystems in both Asia and Africa, and the risk of neglecting these complex systems for the eradication process. More evidence based on well-structured ecological and epidemiological studies at these interfaces are needed to make sure that we understand the role of wild and atypical host species and progress along the eradication path without missing any blind spots. The authors also highlight that PPRV infection is a threat for the conservation of some endangered wild species, which could also help diversify sources for funding the eradication efforts.

The relevance of the global eradication approach is also discussed. For example, instead of large-scale vaccination coupled with monitoring and surveillance, Cameron argues for targeted and adaptive vaccination campaigns informed by real-time data collection, which however assumes the availability of sufficient capacity and resources. Rossiter recommends developing a global and multidisciplinary network linking those implementing field programmes with researchers.

This Research Topic presents some new findings, using a range of methods, from participatory approaches to mathematical modeling and phylogenetics. It also highlights gaps in knowledge, especially the role that atypical species might play in the maintenance of the virus.

Finally, this Research Topic points to the need for continued discussions and reassessments of the global strategy, and for the use of models, along with other scientific methods, to effectively tailor the implementation of this strategy to local contexts (2). Even if PPR is not a zoonosis, its impact on people, and wild and domestic animals, means that a One Health approach is recommended, which would strengthen system thinking around PPR control (3) and would help the integration of disciplines and sectors (Figure 1). Strengthening of wildlife health capacities in the affected regions and globally is important (4, 5) for diseases such as PPR (6) and more widely with other multi-host infections. Wildlife authorities need to be more integrated in the formulation of strategy and policy and in supporting surveillance, especially during the period of verification of freedom from infection and disease at national levels. The establishment of a new One Health Council incorporating United Nations Environment Programme along with WHO, FAO, and OIE is a step in this direction (7).

**Figure 1:**
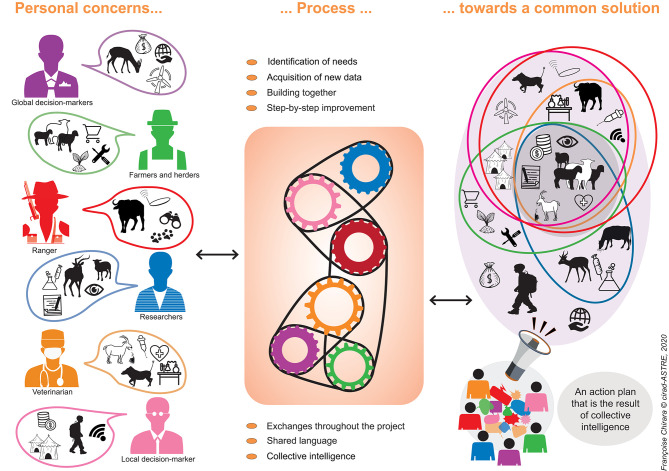
Adapted from (1). Integrated approaches in animal health, CIRAD-ASTRE, *Salon International de l'Agriculture*, Paris, February 2019. This figure illustrates how an integrated approach to PPR needs to be designed in a given context by key players (farmers, veterinarians, rangers, traders, decision makers, etc.) who together characterize the problems and find solutions to be implemented step by step, taking into account the social and economic context, and environmental changes in addition to the animal health aspects.

As the success of the eradication efforts will greatly depend on farmers' willingness to participate in vaccination and surveillance programmes, as demonstrated during the rinderpest eradication programme (8), greater consideration must be given to research in social sciences (9). The achievement of this international programme also requires economic perspectives (10). In this context, public-private partnerships need to be encouraged (11).

The FAO/OIE PPR global control and eradication strategy (PPR GCES) needs a huge financial commitment (12), capacity building and technical support, but also well-funded *ad-hoc* interdisciplinary research that addresses important knowledge gaps for eradication, and effective scientific networks through the Global Research and Expertise Network PPR GREN^1^.

## Author Contributions

All authors have served as editors of the Research Topic. FR has written the draft of the editorial and it was amended and revised by the other authors.

## Conflict of Interest

The authors declare that the research was conducted in the absence of any commercial or financial relationships that could be construed as a potential conflict of interest.

## Footnotes

^1^http://www.fao.org/ppr/news-and-events/news/detail/fr/c/1190842/ (Accessed January 11, 2020).
